# Comparison of Val66met Polymorphism of BDNF gene in patients of bipolar disorder and healthy controls.

**DOI:** 10.1192/j.eurpsy.2024.693

**Published:** 2024-08-27

**Authors:** M. Srikantamurthy, S. Moirangathem, B. Viswanath, M. Purushottam, S. Jain

**Affiliations:** ^1^Department of psychiatry, Orygen youth health, Melbourne, Australia; ^2^Department of psychiatry; ^3^Department of molecular genetics, National Institute of Mental Health and Neurosciences, Bangalore, India

## Abstract

**Introduction:**

The study aims to explore the utility of BDNF Val66Met polymorphism as a potential biomarker in Indian bipolar disorder patients and its correlation with clinical characteristics.

**Objectives:**

Genotyping Val66Met in BDNF gene

Exploring its association with bipolar disorder (BD).

**Methods:**

150 consenting BD patients and matched controls were recruited using a case-control study design. BD severity was assessed using Young’s mania rating scale and the Clinical Global Impression - Severity (CGI-S) scale. BDNF Val66Met polymorphism was identified through real-time PCR after DNA extraction. Data was tested for normal distribution. Genotype frequencies between two groups were compared and the Hardy-Weinberg equilibrium assumptions were tested using Chi-Square tests. Clinical-genotypic associations were explored using the Kruskal-Wallis test and confirmed using hierarchical regression.

**Results:**

Our sample had more males (60%) than females (40%) with mean age of 35.05 years. Most patients had established bipolar disorder and were severely ill (CGI: 38.75, YMRS). Val66Met SNP genotype frequency differed significantly between cases and controls. Val66Val genotype and Val allele were higher in cases. Results consistent with Hardy-Weinberg equilibrium.

**Table 1.** Genotype frequencies of BDNF (rs6265) in cases and controls

GENOTYPE			
	CC	CT	TT
CASES	94(62.6%)	47(31.3%)	9(6%)
CONTROL	71(47.3%)	69(46%)	10(6.6%)
CHI-SQUARE- 7.431	DF(Degree of freedom) - 2	p-value- 0.024

**Table 2.** Dominant genotype frequencies in cases and controls

DOMINANT GENOTYPE		
	CC	CT+TT
CASES	94(62.6%)	56(37.3%)
CONTROLS	71(47.3%)	79(52.6)
CHI-SQUARE-7.125	DF(Degree of freedom)-1	p-value-0.007

**Table 3.** Allelic frequencies of BDNF (rs6265) in BD cases and healthy controls

ALLELIC VARIATION		
	C	T
CASES	235(78.3%)	65(21.6%)
CONTROLS	211(70.3%)	89(29.6%)
CHI-SQUARE-5.032	DF(Degree of freedom)-1	p-value- 0.024

**Conclusions:**

Our study found that Val66Val genotype and Val allele were higher in cases and could be a potential biomarker for bipolar disorder (BD), which is consistent with previous research conducted on the European population. However, further investigations are required to gain a more comprehensive understanding of its impact on BD, including its association with serum BDNF levels, treatment outcomes, and a more diverse study population.

**Disclosure of Interest:**

None Declared

